# Supplementary material for: Virus-Induced galactinol-sucrose galactosyltransferase 2 Silencing Delays Tomato Fruit Ripening
Source: Plants (Basel). 2024 Sep 21;13(18):2650. doi: 10.3390/plants13182650 (PMC11434899; doi:10.3390/plants13182650)
Supplement: Supplementary file 1 [file plants-13-02650-s001.zip › plants-3184446-supplementary/Supplementary Materials.pdf]

Supplementary Materials

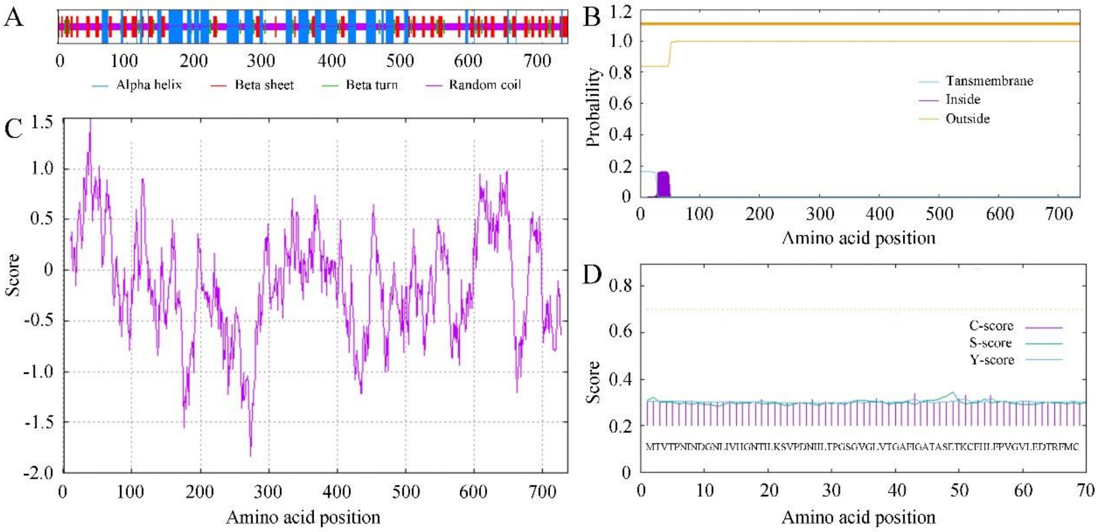

Figure S1. The prediction of secondary structure (A), transmembrane topology (B), hydropathicity of amino acids composition (C) and signal peptides (D) of GSGT2. The C-score indicates raw cleavage site score, S-score indicates signal peptide score, and Y-score indicates combined cleavage site score.

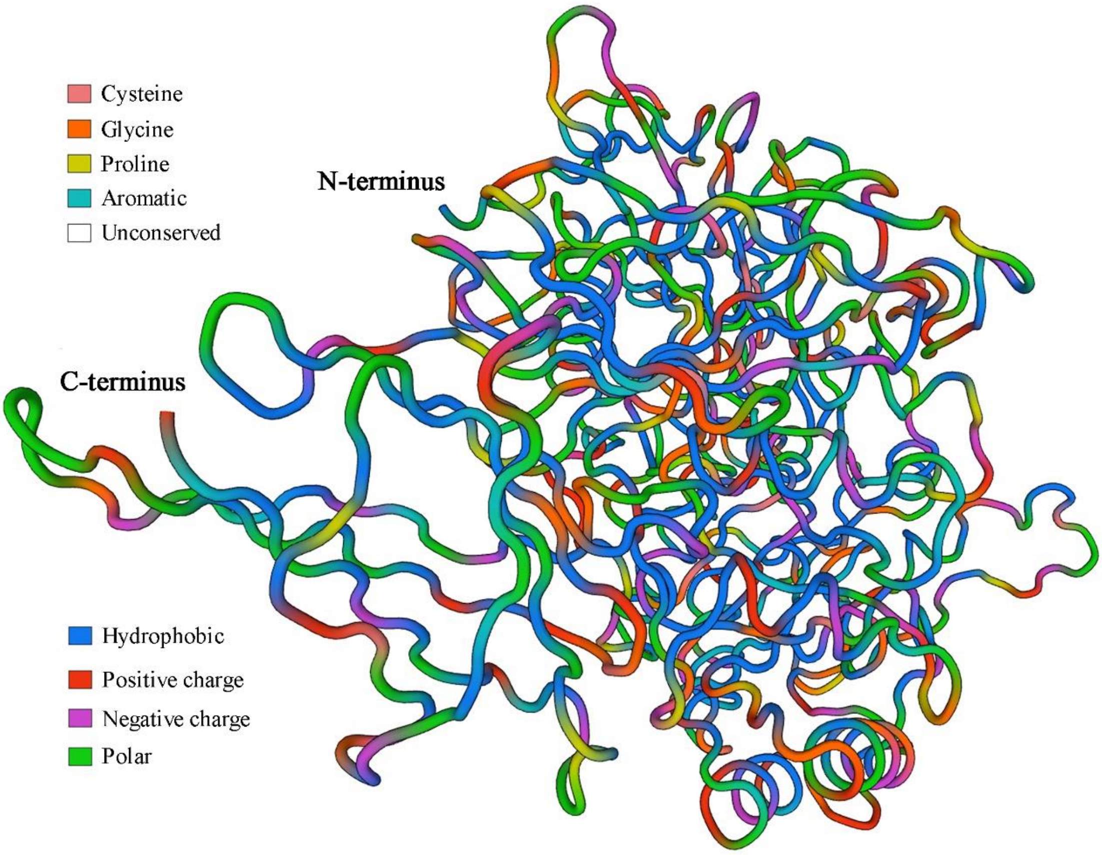

Figure S2. The three-dimensional model prediction of GSGT2. The model is computed by the SWISS-MODEL server homology modeling pipeline which relies on an in-house comparative modeling engine ProMod3. The model quality is described by GMQE (Global Model Quality Estimate) between 0 and 1, with higher numbers indicating higher quality. The GMQE value of this

model is 0.94. Each residue in the alignment is assigned a color if the amino acid profile of the alignment at that position meets some minimum criteria specific for the residue type.

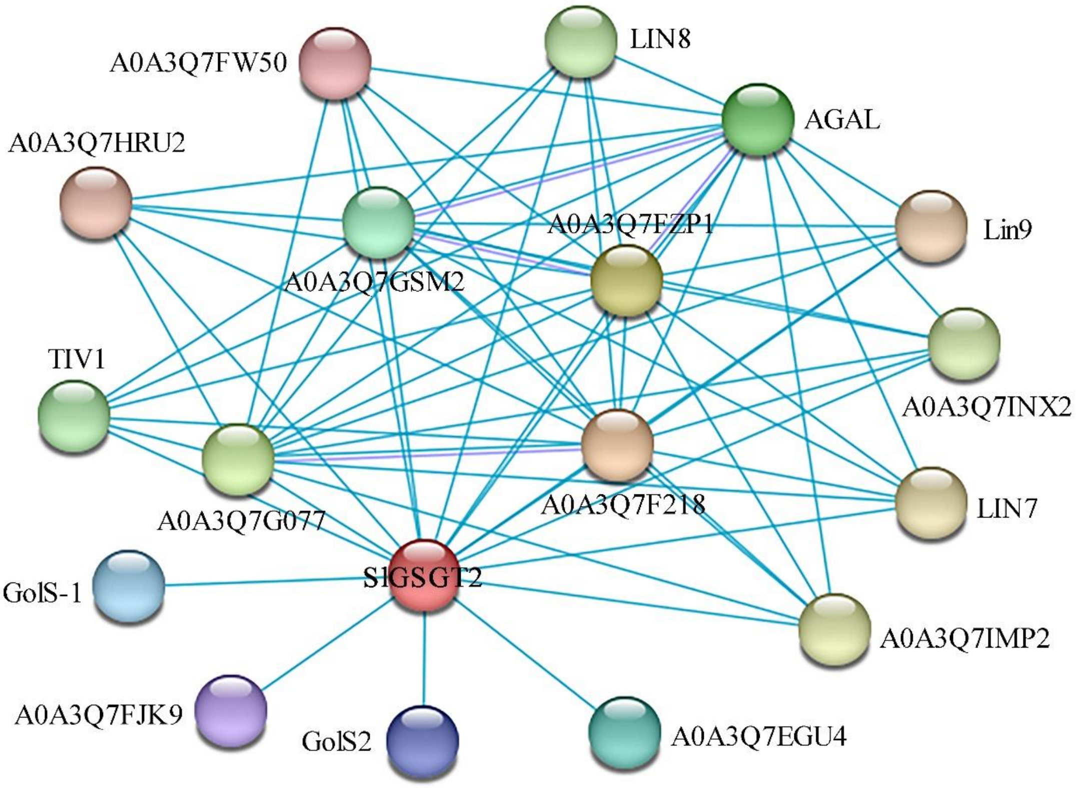

Figure S3. The predicted interaction network of SlGSGT2 in tomato fruit. Network nodes represent proteins, and edges represent protein–protein association. Purple lines indicate that the known interaction is experimentally determined, and blue lines indicate that the interaction is from curated databases. The red node in the network center represents the query protein SlGSGT2. Detailed information on each protein can be found in Table S2.

Table S1. Information on the primers used in this study.

Table S2. Information on the predicted functional partners of SlGSGT2.
